# Knowledge, Attitude, and Intention to Receive the Pertussis Vaccine in Pregnant Women: A Systematic Review

**DOI:** 10.3390/healthcare13233139

**Published:** 2025-12-02

**Authors:** Franciszek Ługowski, Julia Babińska, Aleksandra Urban, Joanna Kacperczyk-Bartnik, Paweł Bartnik, Ewa Romejko-Wolniewicz, Jacek Sieńko

**Affiliations:** II Department of Obstetrics and Gynecology, Medical University of Warsaw, 02-091 Warsaw, Poland

**Keywords:** pertussis, pregnancy, maternal vaccination, Tdap, vaccine hesitancy

## Abstract

**Background:** Pertussis is a respiratory infection that represents a significant threat worldwide, especially for infants. The global incidence of pertussis is on the rise, with 20–40 million cases occurring every year. Maternal vaccination offers protection to newborns and, therefore, is recommended by numerous healthcare organizations. The aim of this study was to systematically assess the level of knowledge regarding pertussis and the pertussis vaccine, as well as the willingness to receive the vaccine among pregnant women, and identify the most significant reasons for vaccine hesitancy among the obstetric population. **Methods:** A systematic literature search was conducted in the Web of Science, Embase, and Scopus databases between 1 April 2024, and 31 July 2024. Our search strategy aimed to identify studies published from 1 January 2014 to July 2024 in order to capture a decade’s worth of the most recent evidence and updates in maternal pertussis vaccination. **Results:** We screened 955 articles altogether, with 11 studies included in the analysis. The general awareness of pertussis infection prior to participation in the study varied from 5% in a study performed in Turkey to 95.9% in the Norwegian population. Moreover, the willingness to receive the vaccine ranged from 11.2% in the Turkish population to 94.8% in the Netherlands. Several statistically important factors affecting the decision have been identified, such as belief in safety and effectiveness, fear of adverse reactions, or healthcare professional recommendation. **Conclusions:** The general awareness regarding pertussis vaccine in pregnant women differs significantly depending on the population studied. However, it remains unsatisfactory even in populations with a high declared level of knowledge if asked specific questions. Presented results may indicate the need for studies on the efficacy of educational interventions for raising awareness about the meaning of pertussis immunization during pregnancy and preventing infection among neonates.

## 1. Introduction

Pertussis is a respiratory infection caused by the human-specific bacteria *Bordetella pertussis*, as well as the related bacteria *Bordetella parapertussis*. Its most common form includes paroxysmal coughs ending with a distinctive prolonged crowing intake of breath [1]. Frequent outbreaks are distinctive of this endemic disease. The disease can occur at any age; however, its course is most severe in infants. In the United States, over 80% of infants with pertussis below the age of 2 months are hospitalized [2]. The global incidence continues to increase, with an estimated number of 20–40 million cases worldwide, according to the World Health Organization (WHO), including 160,700 children under the age of five [3]. The disease can be fatal, with the most significant complications such as pulmonary hypertension, heart failure, and encephalitis [4]. Moreover, there have been numerous reports of macrolide-resistant *Bordetella pertussis* in infants as well, which constitutes a significant threat for neonates globally [5,6]. A study in Germany, conducted prior to common pertussis vaccination, found that 90% of unvaccinated infants and children experienced paroxysmal cough in the course of pertussis, 79%—whooping, 53%—post-tussive vomiting, while fever occurred in 6% of patients [7]. Following the introduction of the universal immunization, there has been a switch in the mode of transmission from child-child to adult-child, which constitutes a major epidemiologic challenge [8]. This mode increases the risk of infection in infants, making pertussis a major cause of infant mortality. Vaccination is one of the primary preventive measures (PPM). Currently, there are two types of vaccines—whole-cell and acellular. The acellular pertussis vaccine (aP) was first introduced in Japan in 1981 [9]. It contains a purified pertussis-related antigen, the amount and concentration of which differ, depending on the manufacturer, which might affect its immunogenicity [10]. The estimated efficacy of aP varies from 70% to 90% in accordance with the targeted population and study design [11,12]. On the other hand, the whole-cell pertussis vaccine (wP) comprises nonviable bacterial cells with major antigens, for instance, pertussis toxin (PT), adenylate cyclase toxin, or filamentous hemagglutinin. The *Bordetella pertussis* bacteria are grown in a liquid medium. The reported efficacy of wP varies from 36% to 98% [10,13,14]. Even though aP is associated with fewer adverse reactions, several studies have shown that it provides a higher risk of pertussis morbidity [15,16]. Vaccinating pregnant women against pertussis has a 90% efficacy in preventing the disease in infants during their first two months of life, until they can be vaccinated [17]. The Joint Committee on Vaccination and Immunization (JVCI, UK) recommends vaccination between 16 and 32 weeks of gestation. On the other hand, the Advisory Committee on Immunization Practices (ACIP) suggests vaccination between weeks 27 and 36 in order to achieve the greatest transplacental transfer of antibodies to the fetus [18,19]. The acellular vaccine is currently the only vaccine recommended as safe for pregnant women [20]. Vaccinating pregnant patients against pertussis with only the acellular vaccine is justified due to its improved safety profile, as it contains purified components rather than whole cells, reducing the risk of adverse reactions for both the mother and the fetus. It is noteworthy that PPMs are crucial for preventing and managing pertussis, especially among pregnant women, and include not only vaccination during pregnancy but also adherence to the basic rules of hygiene, especially after contact with contaminated bodily fluids. Raising awareness of the risk of pertussis infection is of utmost significance, as the very first case of an infant’s death in Poland has been recorded recently [21]. Infant deaths due to pertussis have been reported recently in other European countries as well [22,23]. There are numerous reasons for pertussis resurgence, such as past underidentification of pertussis cases, decreasing immunity after immunization with the acellular vaccines, and a large susceptible population [24]. Furthermore, pathogen evolution has caused more rapid changes in acellular vaccine antigens than other proteins, which causes outbreaks [25]. Assessing pregnant patients’ knowledge about pertussis vaccination during pregnancy can help identify the gaps in understanding, address concerns, and improve vaccination rates, ultimately enhancing protection for both mother and newborn.

The aim of this study was to systematically review the literature and assess pregnant women’s knowledge of pertussis, including infection, preventive measures, complications, attitudes and concerns, and intentions of vaccination. Moreover, our analysis focused on elucidating the most common reasons for vaccine hesitancy among the obstetric population.

## 2. Materials and Methods

### Search Strategy and Selection of Studies

**Figure 1:**
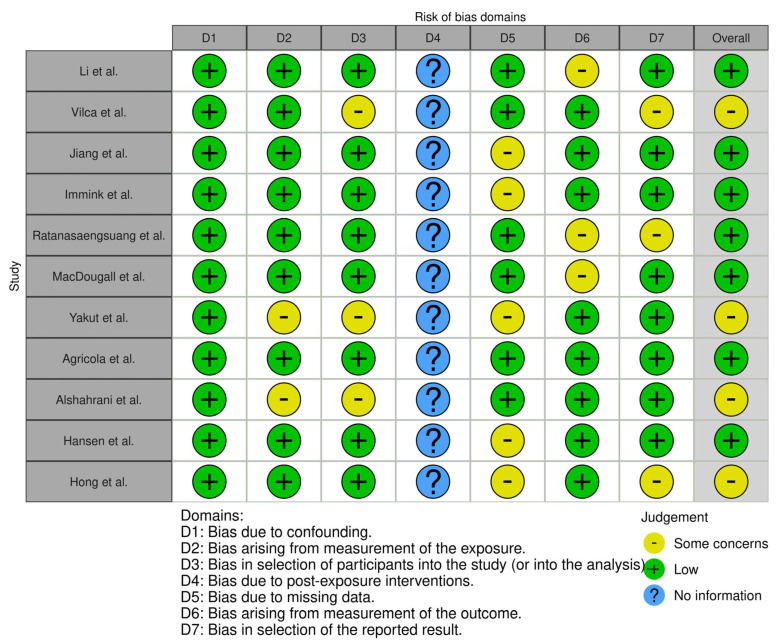
Assessment of quality and risk of bias based on ROBINS-E [26,27,28,29,30,31,32,33,34,35,36].

A systematic literature search was performed in the databases of Web of Science, Scopus, and Embase between 1 April 2024 and July 2024. Our search strategy aimed to identify studies published from 1 January 2014 to 31 July 2024 in order to capture a decade’s worth of the most recent evidence and updates in maternal pertussis vaccination. Although our inclusion period began in 2014, the earliest eligible study meeting our criteria was published in 2016. The review was conducted following the Preferred Reporting Items for Systematic Reviews and Meta-Analyses (PRISMA) guidelines, and the study protocol has been registered in the PROSPERO database with the registration number CRD42024609627. The following search terms and their combinations were used: (“knowledge” OR “attitude” OR “practice”) AND (“pregnant” OR “pregnant women” OR “maternal”) AND (“pertussis”) AND (“vaccine” OR “vaccination”). The search was conducted using both MeSH terms and free-text keywords to ensure comprehensive coverage of relevant studies. Filters were applied to include only studies published in English. Reference lists of included studies were manually screened for any other eligible studies. The defined population was pregnant women, both primiparous and multiparous. The inclusion criteria were cross-sectional studies in English that assessed at least one of the following: knowledge about pertussis, knowledge about the pertussis vaccine, attitude, and willingness to receive the vaccine. The risk of bias in included studies was assessed independently by two researchers (F.Ł. and J.B.) using the modified Downs and Black Checklist for systematic reviews. Only studies with a score of at least ten out of thirteen points possible were included. Only one study achieved ten points [26] by only one researcher; all of the other included studies scored at least eleven, which is considered “excellent”. Additionally, the ROBINS-E (Risk of Bias in Non-Randomized Studies of Exposures) tool was used to evaluate the risk of bias in order to provide a systematic approach to assess the quality and reliability of evidence from the selected studies (Figure 1). The exclusion criteria were other types of studies and studies in languages other than English. This decision was based on both practical considerations, such as the lack of reliable translation resources, and the fact that the selected databases predominantly index high-quality, peer-reviewed literature in English, ensuring consistency and scientific validity across the included studies. Moreover, we excluded studies enrolling non-pregnant individuals. Following the initial screening, the pre-selected studies were further analyzed to assess final eligibility for the systematic review.

Data were extracted independently by two researchers (F.Ł. and J.B.) The following data were extracted: authors’ names, type of article, year of publication, sample size, data collection method, and the results of questions inquiring about information on pertussis infection, primary preventive measures, possible complications, attitude, and willingness to get vaccinated. Due to the heterogeneity of study designs, populations, and outcome measures across the included articles, a quantitative meta-analysis was not feasible. Therefore, we employed a narrative synthesis approach to summarize the findings. Data extracted from each study were categorized by knowledge of pertussis, knowledge of the pertussis vaccine, attitudes toward vaccination, and intention to receive the vaccine. Themes and patterns across studies were identified, and comparisons were made based on geographical regions, healthcare settings, and reported determinants of vaccine hesitancy and acceptance.

## 3. Results

**Figure 2:**
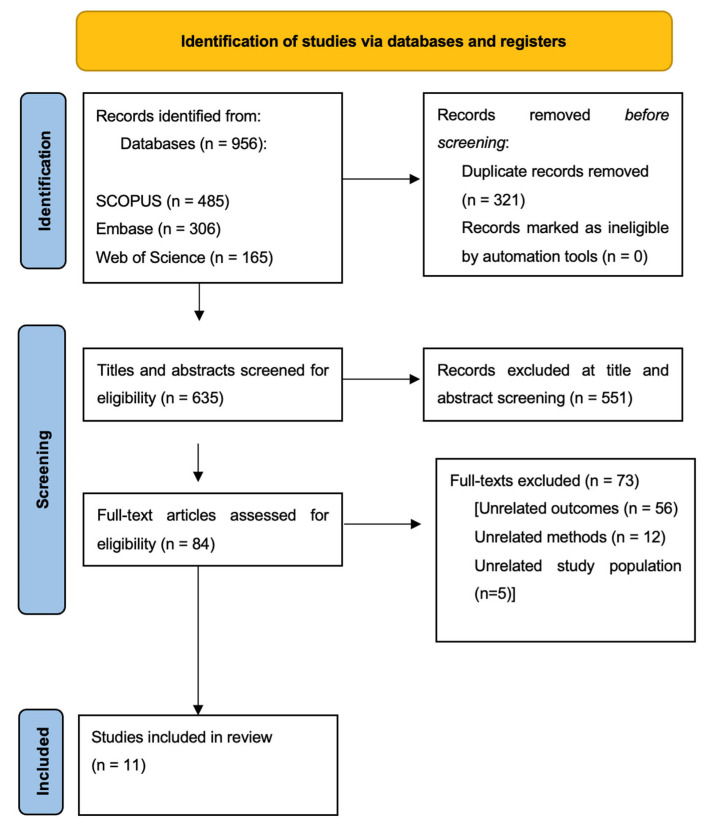
PRISMA flow chart of the screening process.

A total of 956 articles were identified through a systematic review of the literature (Figure 2). After initial screening, 321 duplicates were excluded, and 635 titles and abstracts were further screened to evaluate eligibility, of which 82 were excluded due to language restrictions. A total of 84 publications underwent an in-depth full-text analysis, resulting in 73 studies being excluded from further assessment. Eventually, a total of 11 publications were included in this systematic review.

Across the included studies, several key patterns emerged regarding pregnant women’s knowledge, attitudes, and intentions toward pertussis vaccination.

The general awareness about pertussis varied significantly depending on the population studied and was either very high or high in well-developed countries or was considered low or very low in developing countries. The lowest general awareness occurred in the Turkish population (Table 1) [26], whereas the greatest was among the Norwegian population (Table 1) [35]. The general awareness ratio, based on declaration or according to the results of the distributed questionnaire, exceeded 50% in 6 out of 9 studies that examined this matter [27,31,32,33,35,36]. The three exceptions were the Saudi Arabian study by Alshahrani et al. which reported that 57.9% of respondents were unaware of pertussis infection; the Turkish study by Yakut et al. which showed that 66.2% had not heard of pertussis and 28.6% were not sure; and the Chinese study by Jiang et al. in which only 35.99% of respondents considered themselves susceptible to pertussis [26,29,34]. In our view, even the studies with splendid awareness should be approached cautiously. In the study by MacDougall et al., 32.4% of patients were unaware of the pertussis vaccine. Furthermore, 35.8% of pregnant women could not tell whether maternal vaccination would help prevent the newborn from the disease [32]. Moreover, in the study by Hansen et al., in which the declared awareness of pertussis was 95.9%, 25.8% disagreed that pertussis can be very easily transmitted, 20.2% disagreed that pertussis could be serious in pregnant women, and 55.2% of surveyed women disagreed that their baby could contract pertussis [35].

Ten out of eleven studies included a specific question about whether the respondents would accept the vaccine [26,27,29,30,31,32,33,34,35,36]. The average percent of women willing to receive maternal vaccination was 48.79% ± 27.59. The highest was reported by Immink et al. (94.8%), and the lowest by Yakut et al., 11.2% [26,30]. Four studies showed a willingness rate of over 50% [27,30,35,36]. However, the study by Ratanasaengsuang et al. included two questions. Firstly, respondents were asked if they would like to receive the vaccine unconditionally, and only 46% answered “yes”; however, if asked about receiving the vaccine if a physician recommended it, 81.9% would agree [31]. The study by MacDougall et al. only included a question associated with a doctor’s recommendation, and 89% of pregnant women would agree [32].

Knowledge Gaps: while general awareness of pertussis infection varied widely, specific knowledge regarding disease severity, transmission, and vaccine safety was often insufficient. For example, in Norway, although 95.9% of respondents had heard of pertussis [35], only 30.9% agreed that vaccination was safe during pregnancy. In contrast, in Turkey, only 5% of women had ever heard of pertussis prior to participation [26], indicating a profound knowledge gap in certain regions. Similarly, in Saudi Arabia, the majority of respondents lacked basic information on the nature of pertussis and its vaccine efficacy [34].

Misconceptions about Vaccine Safety: concerns about vaccine safety for the fetus were a frequent barrier to acceptance. In Taiwan, 43.8% of pregnant women declined vaccination due to fear of adverse reactions affecting the fetus [27]. In Italy, 29% believed that the vaccine may harm the fetus [33], while in Saudi Arabia, most participants expressed uncertainty about the safety profile of pertussis vaccination during pregnancy [34].

Influence of Healthcare Provider Recommendations: across nearly all studies, a recommendation from a healthcare provider emerged as one of the strongest predictors of vaccine acceptance. For instance, in Italy, women who received a recommendation from a healthcare provider were 67 times more likely to accept vaccination [28]. Similarly, in China, 35.99% of participants agreed to vaccination when recommended by their physician [29]. In Thailand, willingness to vaccinate increased from 45.5% to 81.9% when linked to physician recommendation [31].

Geographic and Socioeconomic Disparities: both knowledge and vaccine uptake varied significantly between countries and healthcare systems. In the Netherlands, intention to vaccinate reached 94.8% [30], while in Turkey, only 11.2% reported willingness to receive the vaccine [26]. Factors such as education, prior vaccination history, and access to healthcare likely contributed to these variations.

Cultural, Religious, and Moral Beliefs: sociocultural factors contributed to vaccine hesitancy in some populations. In Taiwan and Italy, moral or religious objections were reported [27,33]. In Saudi Arabia, 1.7% of women were discouraged from vaccination by family members [34]. General opposition to vaccination was also reported in several studies [26,34].

Barriers to Vaccination: frequently reported barriers included lack of awareness, fear of side effects, absence of healthcare provider recommendation, misinformation, and sociocultural concerns. In Saudi Arabia, 55.1% of women refused vaccination despite free vaccine availability, and 1.7% were discouraged by family members [34]. In Italy and Taiwan, moral and religious beliefs, along with mistrust in vaccine information, were also identified as contributing factors [27,33].

Correlation Between Knowledge and Uptake: several studies demonstrated that better knowledge was associated with higher vaccine acceptance. In Taiwan, pregnant women who rated pertussis as a highly severe and contagious disease were significantly more likely to accept vaccination [27]. Likewise, in Italy, awareness that maternal vaccination protects newborns doubled the likelihood of vaccine acceptance [28].

Structural and System-Level Barriers: in some settings, systemic issues such as lack of healthcare provider recommendation, vaccine availability, and cost acted as barriers. In Italy, 81% of unvaccinated women reported not receiving any recommendation from healthcare providers [28]. In Saudi Arabia, 55.1% of women refused vaccination even when the vaccine was offered free of charge [34]. Similarly, Jiang et al. identified perceived barriers and benefits as significant predictors of vaccination intention in China (PR = 1.08, 95% CI: 1.04–1.12 and PR = 1.06, 95% CI: 1.03–1.10, respectively) [29].

These findings emphasize the critical importance of healthcare provider engagement, targeted education, culturally adapted messaging, and system-level interventions to improve maternal pertussis vaccination uptake globally.

## 4. Discussion

This review provides a systematic synthesis of recent literature assessing pregnant women’s knowledge, attitudes, and intention to receive the pertussis vaccine across diverse international settings. Rather than focusing solely on descriptive findings, our analysis highlights important thematic patterns and determinants that influence maternal vaccine uptake. The insights from this synthesis underscore not only the variability in awareness and behavior but also critical opportunities for healthcare systems and providers to intervene effectively.

These findings suggest that while general disease recognition may be high in some settings, specific and actionable knowledge remains lacking. This distinction is critical: it implies that public health strategies must move beyond awareness campaigns and instead focus on delivering precise, comprehensible information about disease severity, vaccine safety, and timing. Without this specificity, even women who consider themselves “aware” may remain vulnerable to misinformation or inaction.

The knowledge about the pertussis vaccine was analyzed in ten studies [26,27,28,29,31,32,33,34,35,36]. Most studies reported an unsatisfactory level of knowledge in this matter [22,23,25,27,28,29,30,31]. In the study by MacDougall et al., 32.4% of patients were unaware of the pertussis vaccine’s existence. Furthermore, 35.8% of pregnant women could not tell whether maternal vaccination would help prevent the newborn from the disease [32]. Moreover, the study in Saudi Arabia showed that only 25.2% had heard of the vaccine, only 10.7% knew about safety and potential adverse reactions, and only 18.7% were aware that it can protect newborns [30]. The following are also problems in other studies [26,28,29,33]. Other issues regarding pertussis vaccine awareness presented in the studies included in the review are lack of vaccination counseling with doctors [27,28], lack of trust in the information about the vaccine [27,28], “moral responsibility” defined as religious beliefs, as in some cultures vaccines may be falsely associated with something immoral [29], belief that the vaccine might negatively affect fetal growth [24], or being opposed to vaccination in general [33]. Even the studies that revealed very good declared awareness showed that specific knowledge about the pertussis vaccine is rather low [35,36]. The study in Norway reported that 57% of women did not know if vaccination could increase the risk of birth defects, 30.7% did not know if it could protect newborns from severe disease, and 16% disagreed that vaccination could be harmful to the fetus [35]. On the other hand, Hong et al. reported relatively good awareness, with 65% of women knowing that maternal vaccination can protect the child. Interestingly, the same percentage of respondents knew that not all vaccinations should be avoided during pregnancy [36].

Education level was frequently associated with both knowledge and vaccine intention. Several studies suggested that higher educational attainment correlated with better understanding of pertussis and stronger belief in vaccine efficacy. However, even in populations with high general education levels, gaps in specific knowledge persisted. For instance, many respondents across studies could not correctly identify whether maternal vaccination protects infants or misunderstood its safety profile. This highlights the need for targeted, medically accurate, and accessible communication strategies regardless of baseline education.

The results of our analysis suggest that a physician’s recommendation is of great significance for patients in the decision-making process regarding maternal vaccination. In conclusion, the general willingness to receive the pertussis vaccine is low in most populations; however, it differs significantly according to different studies.

Across multiple studies, one consistent and powerful determinant of vaccine acceptance was the recommendation by a healthcare provider. This highlights the critical, strategic role that clinicians—especially obstetricians, midwives, and family physicians—play in influencing maternal vaccination decisions. Routine integration of vaccine counseling into prenatal care could address both informational and motivational barriers. Importantly, these findings suggest that training healthcare providers to engage in effective vaccine communication may be as impactful as public health campaigns targeting pregnant women directly.

Apart from the willingness to vaccinate, a detailed analysis of the reasons why patients decide or refuse such preventive services is crucial. Particular emphasis should be put on the participation of the attending physician in the process of substantive preparation of the patient to make a conscious decision. All the analyzed studies attempted to establish factors associated with the willingness to receive the pertussis vaccine [26,27,28,29,30,31,32,33,34,35,36]. Li et al. found that a higher likelihood of vaccination was associated with (1) rating pertussis among infants aged 0–3 months as highly severe (*p* < 0.0001), (2) rating prenatal vaccination as effective in preventing neonatal pertussis (*p* < 0.0001), and (3) rating the vaccine as very safe for the fetus (*p* < 0.0001). The same study reported that the most common cause for accepting maternal vaccination was the belief that maternal vaccination could protect the infant from pertussis (35.3%). On the other hand, the most frequent reason for declining the vaccine was the fear of possible vaccine adverse reactions to the fetus (43.8%) [27]. Also, in the Italian study by Agricola et al., a belief that the vaccine is harmful to the fetus was described as a pivotal factor against vaccination [33]. Vaccine safety was also discussed as a crucial factor in the study by Ratanasaengsuang et al. [31]. Jiang et al. reported that perceived barriers (PR  =  1.08, 95% CI: 1.04–1.12, *p* <  0.01) and cues for action or perceived benefits (*PR*  =  1.06, 95% CI: 1.03–1.10, *p* <  0.01) were significantly associated with vaccination intentions. On the other hand, perceived susceptibility, severity, and sociodemographic factors were not statistically important associations [29]. As for the correlation between knowledge and willingness to receive maternal immunization, Vilca et al. found that pregnant women who were aware that the vaccine provides protection to newborns were more likely to receive the vaccine (OR = 2.2, 95% CI, 1.1–4.4). Moreover, the most significant factor associated with willingness to vaccinate in this study was receiving a recommendation from a healthcare professional (OR = 67; 95% CI, 27–201) [28]. It was surprising that the papers analyzed did not indicate a significant contribution of social media in shaping patients’ opinions regarding pertussis vaccination. Hardly ever were other sources mentioned; even if they were, the percentage of answers indicating them was low [33,34]. The review included articles published between 2016 and 2024, when the role of apps and social networks was already well-established and growing year by year. A possible reason for this is the specific study group, pregnant patients, who are nevertheless more willing to rely on the knowledge and opinions of professionals when it comes to pregnancy and medical management than the general population. The impact of the availability and abundance of unverified information in social media on pregnant women’s sense of security and medical decisions is certainly a topic for further research. Other factors associated with a greater chance of vaccination included high income [31], previous DTP vaccination in childhood or adolescence [26], vaccination during prior pregnancies [26], age and educational level [34], availability free of charge [28], belief that vaccination reduces the incidence of pertussis [28], and a recommendation by the Ministry of Health or an international organization [26]. On the other hand, the most frequent reasons behind declining vaccination are lack of awareness about the disease [34], lack of recommendation, general opposition against vaccination [26,34], fear of adverse reactions [26,36], and cost [36].

**Figure 3:**
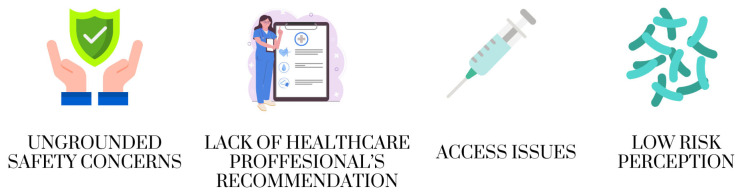
The most common reasons against vaccination for pertussis during pregnancy.

Sociocultural beliefs, including religious considerations, mistrust in pharmaceuticals, and misconceptions about vaccine safety for the fetus, contributed significantly to vaccine hesitancy. In some populations, maternal immunization was seen as potentially harmful or unnecessary. In others, women cited influence from family members or the lack of social norms supporting prenatal vaccination. These findings suggest that public health messaging must be culturally tailored and community-specific to be effective. A summary of the key barriers to maternal anti-pertussis vaccination is illustrated in Figure 3.

Despite the growing recognition of social media as a powerful influence on public health behavior, its role in shaping attitudes toward maternal pertussis vaccination was only minimally addressed in the studies included in this review. Most participants cited healthcare providers or traditional media as their primary information sources. This limited reporting may be due to several factors. First, survey instruments used in the studies may not have included specific questions about social media, reflecting a possible underappreciation of its relevance at the time of data collection. Second, pregnant individuals may actively prefer trusted medical sources over social media during pregnancy, a period often associated with heightened caution and information-seeking behavior oriented toward professional guidance. Nevertheless, the pervasive presence of social media in modern life suggests an untapped opportunity for public health campaigns and clinicians to leverage these platforms to disseminate accurate, culturally sensitive information. Future research should systematically examine the content, reach, and credibility of social media messaging on maternal vaccination to inform targeted intervention strategies.

**Figure 4:**
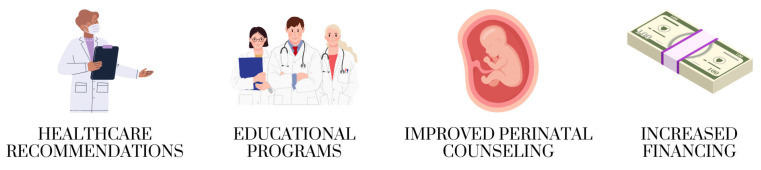
Key strategies to increase the uptake of the maternal pertussis vaccine.

Although the analyzed articles do not address this issue, the undeniable role of national scientific societies and organizations in providing reimbursed and widely accessible vaccinations, as well as in educating the public and countering misinformation about vaccines, cannot be overlooked. We hypothesize that the varying levels of knowledge and differences in attitudes towards pertussis vaccination are influenced by the health policies implemented differently by individual countries. Moreover, discrepancies in guidelines and the qualifications of medical staff must be taken into consideration, as the review indicated that physicians play a crucial role in the patient information process [37]. A summary of strategies to enhance maternal vaccination is presented in Figure 4.

The COVID-19 pandemic, which overlapped with the publication timeline of several studies included in this review, may have influenced public attitudes toward vaccination more broadly. Although none of the included studies explicitly investigated the pandemic’s impact on knowledge or willingness to receive the Tdap vaccine during pregnancy, it is plausible that heightened attention to infectious diseases and increased exposure to vaccine-related messaging affected maternal perceptions and behaviors. On one hand, the pandemic may have improved general health literacy and trust in preventive measures, particularly among individuals closely following public health recommendations. On the other hand, widespread misinformation and growing vaccine hesitancy observed during the COVID-19 era could have negatively influenced attitudes toward maternal immunization. The heterogeneity in vaccine acceptance observed in studies conducted post-2020 (e.g., high acceptance in the Netherlands vs. low acceptance in China and Saudi Arabia) may reflect complex regional and sociocultural dynamics shaped in part by pandemic-related discourse. Future research should explicitly explore how the COVID-19 experience has altered maternal attitudes toward Tdap and other prenatal vaccinations, as understanding these shifts is critical for optimizing future immunization strategies and countering misinformation.

To translate these findings into action, several practical interventions can be considered. First, integrating pertussis vaccination counseling into routine prenatal care—supported by standardized training for obstetricians, midwives, and family physicians—can ensure consistent messaging and increase patient trust. Second, national immunization programs should prioritize making the Tdap vaccine widely accessible and cost-free, particularly in low- and middle-income countries where access remains a barrier. Third, culturally tailored educational materials that directly address common misconceptions—such as vaccine safety or its effectiveness in protecting newborns—should be developed and distributed through both in-person and digital channels. Finally, leveraging mobile health (mHealth) tools and social media campaigns that align with evidence-based guidelines can extend the reach of accurate information, especially to younger and digitally connected populations. These combined strategies could meaningfully reduce vaccine hesitancy and support informed maternal decision-making.

This systematic review has several limitations that should be acknowledged. First, only studies published in English were included, which may have led to the exclusion of relevant research published in other languages. However, the selected databases (Web of Science, Scopus, and Embase) primarily index high-quality, peer-reviewed literature in English, which helps ensure scientific rigor. Second, although our search strategy covered studies published from 2014 to mid-2024 to capture a decade of recent evidence, the earliest included study dates from 2016 due to a lack of eligible publications from 2014–2015. Third, most studies relied on self-administered questionnaires, including online formats, which are susceptible to recall bias and social desirability bias. Moreover, we used Web of Science, Scopus, and Embase, which offer substantial overlap with MEDLINE/PubMed and cover a broad range of peer-reviewed journals. This approach was intended to ensure the inclusion of high-quality studies while allowing for a systematic and reproducible screening process. Furthermore, response rates were not consistently reported across studies, and in some cases, implausibly high rates were presented without supporting details. This raises concerns about potential response bias, as the views of more health-conscious or engaged participants may be overrepresented. Importantly, the heterogeneity in the methodologies of the data acquisition in the analyzed studies, particularly the reliance on self-completed questionnaires, may lead to erroneous conclusions. For example, there is no differentiation between the vaccine types in the questionnaires, probably because they are unknown to respondents, people without a medical background. Additionally, there is a disparity in ethnicity and population sizes across the analyzed cross-sectional studies. Moreover, the findings may reflect data available only up to a certain date, which may not include the latest research or developments in the field, due to the dynamics provided by the availability of emerging information on the controversial and widely discussed topic of vaccination today. Lastly, the included studies varied in terms of geographic settings, sample sizes, and data collection tools, which limited our ability to perform a quantitative synthesis and may affect the generalizability of the findings.

## 5. Conclusions

Pregnant women’s knowledge about pertussis and maternal vaccination, as well as their willingness to receive the vaccine, differs significantly depending not only on the population studied but also on the methodology of the study. However, even in populations with a high declared knowledge, the ratio of correct answers to more specific questions remains unsatisfactory. In this systematic review, we depict the most important factors that affect pregnant women’s attitudes toward the pertussis vaccine. In the presented study, we would like to emphasize the crucial role of the attending physician, not only in providing information about the availability of vaccinations but also in the process of equipping patients with medical knowledge, enabling them to make an informed decision about vaccination. According to the presented literature review, the reluctance to vaccinate often stems primarily from fears of adverse reactions or misconceptions about the vaccine’s effectiveness. Many of these beliefs are not supported by scientific research, and even a brief discussion with the patient could lead to a change in decision. Therefore, educational interventions are still needed in both developed and developing countries, not only among patients but also among medical specialists. These findings underscore the pivotal role of healthcare professionals in promoting maternal immunization. Physicians, midwives, and other prenatal care providers are uniquely positioned to offer evidence-based guidance, dispel misconceptions, and proactively recommend the Tdap vaccine during pregnancy. Even brief yet targeted discussions between patients and providers can significantly enhance vaccine uptake. Therefore, integrating routine vaccine counseling into antenatal care protocols and strengthening provider communication skills should be a strategic priority. Addressing these gaps through training, clear clinical guidelines, and public health policy will be crucial for improving maternal vaccination rates and safeguarding infant health. Future research should prioritize standardized, cross-culturally validated survey tools to allow better comparisons and pooled analyses. Second, more studies are needed on the impact of digital information sources and the post-COVID information landscape. Third, public health strategies should focus on integrating Tdap counseling into routine antenatal care, training providers in vaccine communication, and addressing misinformation through culturally adapted outreach.

## Figures and Tables

**Table 1:** Data extraction results.

	Authors	Publication Year	Country	Study Population	Data Collection Method	Assessed Areas		
						Knowledge of Pertussis	Knowledge of Vaccine	Attitude
1.	Li et al. [27]	2020	Taiwan	1809 pregnant women who had received prenatal care	Self-reported questionnaire at eight maternity hospitals	48% considered pertussis a highly severe and highly contagious disease, which correlated with willingness to take the vaccine (*p* < 0.0001).	62.58% considered maternal pertussis vaccination very effective, which correlated with willingness to take the vaccine (*p* < 0.0001).	The two most frequently selected reasons for accepting the vaccine were (1) belief that maternal Tdap vaccination could protect the infant from pertussis (35.3%) and (2) physician recommendation of prenatal Tdap (33.5%). The two most common reasons for declining the vaccine were (1) fear of possible vaccine adverse reactions to the fetus (43.8%) and (2) believing pertussis is not a severe disease among newborn infants (18%).
2.	Vilca et al. [28]	2020	Italy	743 pregnant women at least 32 weeks of gestation	Self-reported questionnaire at three care centers	Not analyzed	51% were aware that maternal vaccination confers protection to newborns, and 17% believed that the pertussis vaccine can cause the disease in mother and infants.	For unvaccinated women, the main reason against maternal immunization was, “Vaccination was not recommended by any health-care provider (HCP)” (81%). As for the facilitators among vaccinated women, the main reason for accepting vaccines was “I want to protect my baby” (82%).
3.	Jiang et al. [29]	2024	China	564 pregnant women	Self-administered questionnaire in eleven hospital-based maternity units	Only 35.99% considered themselves susceptible to pertussis infection (*p* < 0.001).	Only 35.99% believed that vaccination can reduce the incidence of pertussis and its severity and that it will not affect maternal or fetal health (*p* < 0.001).	Intention to receive maternal vaccination was 36%. 35.99% agreed to take the vaccine because their physician recommended it (*p* < 0.001)
4.	Immink et al. [30]	2023	The Netherlands	1377 pregnant women	Online questionnaire	Not analyzed	Not analyzed	Intention to receive the vaccine was 94.8%. Nulliparous women had a significantly higher mean score for risk perception of pertussis susceptibility in their baby (3.1 vs. 2.9, *p* = 0.039) and a lower mean score for feeling a barrier in being vaccinated against combined vaccine components (2.5 vs. 3.0, *p* = 0.026).
5.	Ratanasaengsuang et al. [31]	2023	Thailand	387 pregnant women aged >18 and >20 weeks of gestation	Self-administered questionnaire	The mean score of knowledge about pertussis and the vaccine among participants was 11.8 ± 2.1, and 259 women (66.9%) correctly answered at least 10 out of 20 questions.	Analyzed altogether with knowledge of pertussis.	45.5% expressed intention to receive the pertussis vaccine, and 52.2% were uncertain. The majority of participants had positive attitudes toward pertussis vaccination during pregnancy. 284 participants (73.4%) believed that maternal vaccination would protect their babies from pertussis, and 261 women (67.4%) strongly believed that vaccination during pregnancy was safe.
6.	MacDougall et al. [32]	2016	Canada	346 pregnant women	Self-administered questionnaire	90.2% of participants had heard of pertussis. The mean number of correct answers to the 19 knowledge questions was 10.65 (95% confidence interval 10.28–11.01).	45.1% “agreed” that maternal vaccination offers protection to newborns, and 17.1% “strongly agreed”.	72.3% of participants agreed or strongly agreed that it was vital for children to be immunized against pertussis by 6 months of age, and 20.5% neither agreed nor disagreed with this statement. While attitudes about receiving the pertussis vaccine during pregnancy were generally favorable, many women neither agreed nor disagreed with these attitudinal statements.
7.	Yakut et al. [26]	2019	Turkey	465 pregnant women	Self-administered questionnaire at an outpatient clinic	In this study, only 24 (5%) participants had heard of pertussis, and only 26 (5.5%) knew that babies can contract pertussis.	Knowledge about the safety of the pertussis vaccine offered to babies was shown to have a positive influence on pertussis vaccination acceptance during pregnancy.	The acceptance rate of the pertussis vaccine was 11.2%. Participants who had a history of pertussis vaccinations during their adolescence or previous pregnancies were significantly more likely to accept a pertussis vaccination (*p* = 0.01 and *p* = 0.013, respectively).
8.	Agricola et al. [33]	2016	Italy	347 women: 164 pregnant women before the 27th week of gestation and 183 postpartum women within 30 days after delivery	Online questionnaire	Regarding knowledge about pertussis risks, nearly 35% of respondents did not know that infants <1 y of age represent the age group with the highest risk of infection.	Regarding knowledge of pertussis vaccination, more than half of the study population answered “undecided” to the specific questions. Among the analyzed population, 29% considered the vaccine as harmful for the fetus.	In the population of pregnant women, 34 (21%) expressed their willingness to vaccinate for pertussis during pregnancy. When asked if they would receive pertussis immunization during the current or a future pregnancy if recommended by an HCP, almost 34% stated the intention of getting the vaccine. Almost 48% declared to be uncertain, and more than 18% stated that they would not get the vaccination, even if they had received a recommendation from an HCP.
9.	Alshahrani et al. [34]	2023	Saudi Arabia	401 pregnant women aged more than 18 years	Interview questionnaire	It was reported that most of the participants ignored basic information about the nature of pertussis disease or its vaccine efficacy.	Most of the respondents doubted the safety of the pertussis vaccination during pregnancy. The majority did not know about the expected adverse reactions or safety levels of the pertussis vaccine during pregnancy or for their children.	We noted that 28 (7%) of the participants were recommended to have the pertussis vaccine, mainly by doctors and healthcare professionals (HCP), whereas seven (1.7%) were discouraged, mainly by relatives and friends, from taking it. Availability of the vaccine at no cost did not change the opinion of 55.1% of participants who refused to be vaccinated during pregnancy.
10.	Hansen et al. [35]	2024	Norway	1148 pregnant women 20–40 weeks of gestation	Online questionnaire	A total of 1101 (95.9%) of the women had heard of pertussis, while 47 (4.1%) women had not heard of or did not know whether they had heard of pertussis.	53.3% were aware that maternal vaccination protects the infant against pertussis. Only 30.9% agreed that it is safe for pregnant women to receive it.	Women who agreed that maternal pertussis vaccination may increase the risk of birth defects or increase the risk of complicated pregnancy were less likely to accept vaccination against pertussis during pregnancy than were women who disagreed. Furthermore, women who disagreed with the statement “I worry that my newborn child will get pertussis” were significantly less likely to accept pertussis vaccination during pregnancy than were women who agreed.
11.	Hong et al. [36]	2023	Singapore	252 pregnant women	Self-administered questionnaire	Only 14% of women knew that the number of pertussis cases is on the rise. 52% of respondents considered pertussis more severe in newborns.	35% of women believed that vaccines should be avoided in pregnancy. 65% were aware that maternal immunization can protect the child.	There was a generally positive attitude towards prenatal vaccination, with 63% of women being in favor of vaccination.

## Data Availability

No new data were created or analyzed in this study. Data sharing is not applicable to this article.

## Abbreviations

The following abbreviations are used in this manuscript:

Acellular pertussis vaccine—aP; Advisory Committee on Immunization Practices—ACIP; Health-care provider—HCP; Primary preventive measures—PPM; Whole-cell pertussis vaccine—wP; World Health Organization—WHO; The Joint Committee on Vaccination and Immunization—JVCI.
